# Contribution of Food, Energy, Macronutrients and Fiber Consumption Patterns to Obesity and Other Non-Communicable Disease Risks in the Indonesian Population

**DOI:** 10.3390/nu17091459

**Published:** 2025-04-26

**Authors:** Fifi Retiaty, Nuri Andarwulan, Nurheni Sri Palupi, Fitrah Ernawati, Renata Kazimierczak, Dominika Średnicka-Tober

**Affiliations:** 1 Department of Food Science and Technology, Faculty of Agricultural Technology, IPB University, IPB Dramaga Campus, Bogor 16680, West Java, Indonesia; fifi007@brin.go.id (F.R.); hnpalupi@apps.ipb.ac.id (N.S.P.); 2 Center for Public Health and Nutrition Research, National Research and Innovation Agency, Cibinong Science Center, Bogor 16680, West Java, Indonesia; fitr043@brin.go.id; 3 Southeast Asian Food and Agricultural Science and Technology (SEAFAST) Center, IPB University, IPB Dramaga Campus, Bogor 16680, West Java, Indonesia; 4 Department of Functional and Organic Food, Institute of Human Nutrition Sciences, Warsaw University of Life Sciences, Nowoursynowska 159c, 02-776 Warsaw, Poland; renata_kazimierczak@sggw.edu.pl

**Keywords:** food pattern, diet, obesity, NCD, Indonesia

## Abstract

Background: Obesity, characterized by excess body fat, has been recognized as one of the main global health problems of the current times. This article, based on the data from the Cohort study of risk factors for non-communicable diseases in Indonesia (FRPTM), aims to analyze the food consumption patterns and their association with the risk of obesity and related non-communicable diseases (NCDs) in the Indonesian population. Methods: The article presents data collected from 867 respondents aged 25 years and above observed for 5 years: 2011, 2013, 2015, 2017 and 2019. It includes sociodemographic characteristics, consumption (1 × 24-h recall), anthropometry, and biomedical data (lipid profile, blood glucose, blood pressure). Results: The study identified cereals as the food group consumed in the largest amount and the largest contributor to energy, protein, carbohydrates and fiber intake. The fats and oils group exceeded the recommended intake, while vegetable and fruit consumption, and consequently the fiber intake, were far below the recommendations. The energy and macronutrient intake, and the percentage of respondents with excessive intake levels, were consequently increasing during the studied years. The consumption patterns were associated with the incidence of obesity, which increased from 43.9% in 2011 to 69.9% (central obesity) and 67.9% (BMI-based obesity) in 2019. Conclusions: The dynamics of the consumption patterns clearly impacted the obesity prevalence. At the same time, the NCDs biomarkers measured remained relatively stable despite increasing obesity and macronutrient intake over the study period. The study provides important insights into diet-related risks for obesity in Indonesia, with a potential to inform public health policies and relevant intervention strategies.

## 1. Introduction

Obesity, characterized by excess body fat, has been recognized as one of the main global health problems of the current times, with its prevalence reported to be dynamically increasing from year to year, including in lower- to middle-income countries [1]. In 2022, 43% of adults aged 18 years and over were overweight, and 16% were obese [2]. Obesity is a risk factor for a number of non-communicable diseases (NCDs), which impact 70% of deaths worldwide, with this figure being predicted to continue to increase in the next decades. Globally, the most common NCDs include cardiovascular disease (CVD) and type 2 diabetes [3]. One of the targets set in the Sustainable Development Goals (SDGs) is to reduce premature deaths from non-communicable diseases by one-third by 2030 [4].

The unbalanced consumption patterns, defined as the combination of foods that individuals and population groups consume, are among the main risk factors for obesity, and the associated NCDs biomarkers, such as increased blood pressure and dyslipidemia [5,6,7]. The macronutrient intake, but also the intake of energy and fiber, depending on the consumption patterns, has previously been suggested to be associated with the incidence of certain NCDs [8,9].

In Indonesia, which is the world’s fourth most populous country, the prevalence of obesity in 2018 was estimated as 21.8% and increased to 23.4% in 2023 [10,11]. At the same time, the mortality rate due to obesity and other major NCDs risk factors has been on the rise in recent decades. For instance, CVDs were estimated to cause over 30% of deaths [12,13], giving Indonesia a fourth position worldwide with the highest rates of CVD-related mortality [13,14]. Although these alarming figures were clearly changing in tandem with a dietary transition [15], yet relatively few studies have looked into the associations between dietary intakes and patterns, and certain NCDs risk factors for the Indonesian population [16,17,18,19].

This article, based on the data from the Cohort study of risk factors for non-communicable diseases in Indonesia (FRPTM), aims to analyze the food consumption patterns of Indonesians and their association with the prevalence of obesity, as an early indication of other non-communicable diseases, including diabetes mellitus (DM), dyslipidemia, hypertension and coronary heart disease (CHD), with a potential to inform public health policies and to serve as a basis for relevant preventive and intervention strategies.

## 2. Materials and Methods

### 2.1. Study Design

This study used a quantitative method with a Secondary Data Analysis approach, using data from the Cohort Study of Non-Communicable Disease Risk Factors (FRPTM) in 2011, 2013, 2015, 2017 and 2019, conducted by the Health Research and Development Agency, Ministry of Health of the Republic of Indonesia. The FRPTM is a study with a prospective cohort design that examines various risk factors related to NCDs. FRPTM study (2011–2019) has been approved by the Health Research Ethics Commission of the Health Research and Development Agency, Ministry of Health of the Republic of Indonesia, with approvals no. KE.01.08/EC/485/2011 dated 10 August 2011, no. LB.02.01/5.2/KE.215/2013 dated 30 May 2013, no. LB.02.01/5.2/KE.135/2015 dated 9 March 2015, no. LB.02.01/2/KE.108/2017 dated 27 March 2017, no. LB.02.01/2/KE.102/2019 dated 8 April 2019.

### 2.2. Study Subjects

The population in this study were adults aged 25 years and above who participated in the Cohort study of risk factors for non-communicable diseases in Indonesia (FRPTM, 2011–2019). A purposive respondent sampling was applied, with the following eligibility criteria: respondents (both women and men) for whom all the data necessary for the purpose of this study’s objectives were available, who consequently participated in the study since 2011 (baseline). This study included 867 respondents from FRTPM observed for 5 years: 2011, 2013, 2015, 2017, and 2019.

### 2.3. Data Collection and Measurements

The data analyzed in the study included sociodemographic data (age, gender, education, occupation), consumption data (1 × 24-h recall), anthropometry (body weight (BW), body height (BH), waist and abdominal circumference), biomedical data (blood lipid profile and blood glucose) and blood pressure data (systolic and diastolic). Sociodemographic data were collected using a questionnaire based on the WHO STEPS instrument (WHO 2024) [20]. Subjects were classified as hypertensive if their systolic blood pressure was ≥140 mmHg or diastolic blood pressure was ≥90 mmHg in two measurements with a 5-min interval. Anthropometric measurements included standard body mass index (BMI) and waist/abdominal circumference. The BMI was calculated as BW in kilograms divided by the square of BH in meters. Subjects were classified as overweight if their BMI was ≥23 kg/m^2^ and as obese if their BMI was ≥25 kg/m^2^, according to the WHO guidelines for Asian populations [21]. Central obesity was recorded if the waist circumference was >80 cm in women and >90 cm in men [21]. The diabetes mellitus (DM) status was determined by fasting blood glucose ≥ 126 mg/dL and blood glucose 2 h postprandial ≥ 200 mg/dL [22]. Dyslipidemia was determined based on the NCEP criteria (ATP III). Subjects with total cholesterol levels of ≥200 mg/dL, LDL cholesterol (LDL-C) levels of ≥130 mg/dL, triglyceride levels of ≥150 mg/dL, or HDL cholesterol (HDL-C) levels < 40 min mg/dL in men and <50 mg/dL in women were classified as dyslipidemic [23]. The LDL-C/HDL-C ratio value > 2.5 was considered a risk factor for hypertension, dyslipidemia and diabetes [24].

### 2.4. Data Analysis

The food consumption data were arranged based on the food group classification according to the ASEAN Food Composition Table [25] and analyzed using the Wilcoxon Paired Test, with *p* values below 0.05 considered statistically significant. Nutrient intake data were analyzed using the Indonesian Food Composition Table (TKPI) [26], the ASEAN Food Composition Table, and the Nutri Survey [27]. The status (below RDA/appropriate/above RDA) was based on the recommendations of the Institute of Medicine (IOM) [28]. The obesity status based on the Body Mass Index (BMI) and the central obesity status were calculated using WHO guidelines [21]. The blood pressure, blood glucose, and lipid profile status referred to the limits set in the WHO guidelines [29].

## 3. Results

### 3.1. Sociodemographic Characteristics of Participants

The socio-demographic characteristics analyzed within the study included age, gender, education, and occupation. This study consisted of 867 respondents who were observed for 5 years. The age groups in this study consisted of adults aged 25 years and above, both females (67.7%) and males (33.3%). The most common occupation of respondents was a housewife/assistant (53.3%), and the least common occupation was a farmer (0.1%). The most common education level of respondents was high school (34.3%), and the least common was no school (1.4%).

### 3.2. Consumption Patterns

Consumption patterns during the 5 years of observation presented in Table 1 show that cereals and their processed products were the food group consumed in the greatest amount. In nearly every year of the observation, 100% of respondents consumed this food group, with the largest average of 259.61 ± 111.36 g in 2011. However, it was still slightly below the expected food pattern (PPH), with the recommended daily intake of 269 g. Fats and oils also belonged to the food groups consumed by almost 100% of respondents in each year of observation, with the largest consumption of 78.62 ± 71.68 g in 2019, far exceeding the expected food pattern (21 g). The main source of animal protein in the participants’ diet was meat and processed meat, consumed by 58.24–74.27% of respondents, with the highest average consumption level of 71.68 ± 76.41 g in 2019. Nuts and their processed products were consumed by 79.58–87.19% of respondents, with the highest average of 94.19 ± 84.36 g in 2017, far exceeding PPH (37 g) [30]. The “sugar, syrup, and confectionery” group was consumed by 58.36–83.27% of respondents, with the highest average consumption of 34.19 ± 26.48 g in 2019. This average did not exceed the recommended limit of the Ministry of Health, which is 50 g/day. The vegetables were consumed by 83.73–90.19% of respondents, with the highest average consumption of 68.08 ± 58.82 g in 2017, while fruits and their processed products were consumed by 42.67–55.59%, with the highest average consumption of 85.88 ± 121.56 g in 2017. Vegetable and fruit consumption was still far below PPH (262 g). The non-alcoholic beverage group was consumed by 84.77–92.50% of respondents, with the highest average of 471.05 ± 411.44 g in 2019. Looking into the consumption dynamics in the studied period (between 2011 and 2019), there was a drop in the consumption of cereals, and a significant increase in the consumption of fats and oils, sugars, syrup and confectionery, non-alcoholic beverages, starchy roots and tubers, and animal-sourced products such as meat, eggs, and milk and dairy. At the same time, there was a significant increase in drinking water consumption, and a slight increase in vegetables and fruits consumption (Table 1).

### 3.3. Energy, Macronutrients and Fiber Intake

**Figure 1:**
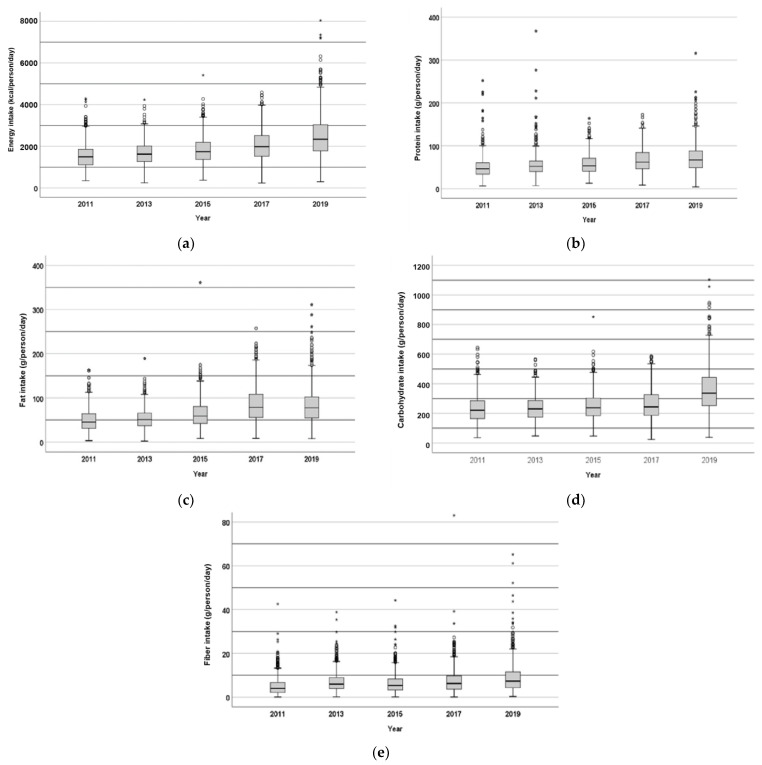
Daily intake of energy (kcal/person/day) (**a**), protein (**b**), fat (**c**), carbohydrates (**d**), and fiber (**e**) (g/person/day) by the population of 867 respondents from FRTPM study in Indonesia during five years of observation (2011–2019).

**Figure 2:**
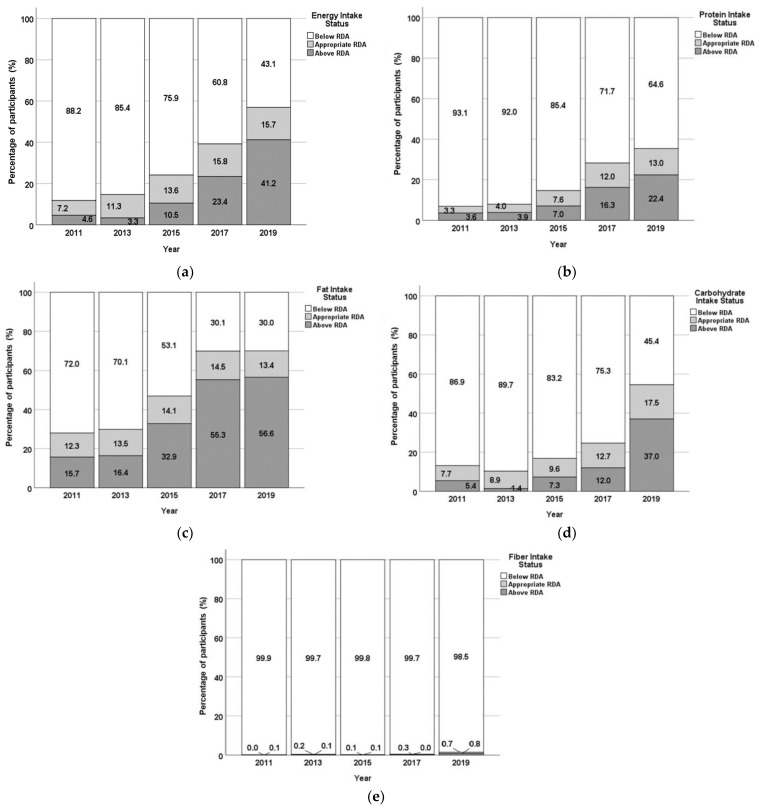
Energy (**a**), protein (**b**), fat (**c**), carbohydrates (**d**), and fiber (**e**) intake status of the population of 867 respondents from FRTPM study in Indonesia during five years of observation (2011–2019): percentage of participants with intake levels below RDA/appropriate/above RDA.

The results on the energy, macronutrients and fiber intake are presented in Figure 1 and Figure 2. The average energy intake showed an increasing trend within the period under investigation (2011–2019), but it was still below the adequate intake of 2250 kcal, except for 2019. At the same time, the percentage of participants with an excessive energy intake status has largely increased in the observed period, from 4.6% in 2011 to 41.2% in 2019.

The average protein intake also increased during the studied period, although it was still below the RDA (60 g), except for 2019. At the same time, the percentage of respondents with adequate or too high protein intake was consequently increasing during the studied period, reaching 13% (appropriate intake) and 22.4% (above RDA) in 2019.

Except for 2017 and 2019, the average fat intake was also below the RDA (65 g). However, as reported for total energy and protein, the percentage of participants with excessive fat intake has largely increased during the 5 years of observation, exceeding 55% of the studied population in 2017 and 2019.

The carbohydrate intake was also increasing during the course of the five years, but the average value was still below the RDA (360 g). At the same time, the percentage of participants showing an excessive carbohydrate intake appears to have been increasing from 2013 to 2019, with the largest increase between 2017 and 2019 (from 12% to 37%).

The average fiber intake was far below the RDA (32 g), with almost 100% of the studied population not reaching adequate intake levels.

### 3.4. Contribution of Food Groups to Nutrient Intake

The results on the contribution of food groups to nutrient intake are shown in Table 2. The cereals and processed cereal-based foods had the largest contribution to energy (47.71%), protein (37.20%), carbohydrates (71.57%), and fiber (28.03%) intake. The fats and oils group is the second largest contributor to energy intake (16.78%) and contributes the most to fat intake (50.69%). Other food groups that bring fat intake contribution of more than 10% are meat and processed meat-based products (14.88%) and nuts, seeds, and their processed products (10.56%).

Food groups that contribute quite significantly to protein intake are also nuts, seeds, and their products (18.02%) and meat and processed meat-based products (16.4%). Other animal protein source food groups, such as fish, shellfish, and other aquatic animals, contribute 9.56%, and eggs and their processed products contribute 7.11%. The average carbohydrate intake has increased during the 5 years of observation, allegedly due to the consumption of sugars from the high-sugar non-alcoholic beverage group, such as sweet drinks made at home, manufactured sweet drinks, and carbonated drinks. The sugar, syrup, and confectionery food group contributes to carbohydrate intake by 8.04%. Food groups that contribute to fiber intake are vegetables and their processed products (18.54%), nuts, grains, and their products (17.82%), and fruit and processed products (17.67%).

### 3.5. Obesity as an Early Indication of Non-Communicable Diseases

**Figure 3:**
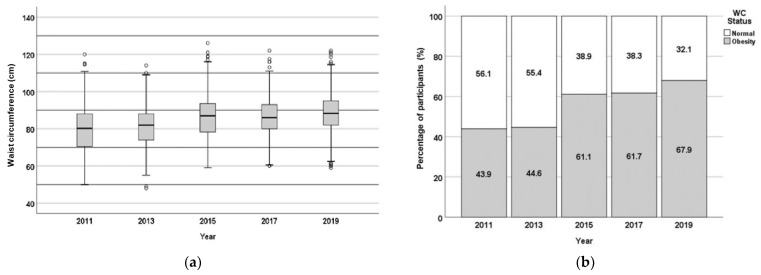
Central obesity status of the population of 867 respondents from FRTPM study in Indonesia during five years of observation (2011–2019), (**a**)—boxplot (waist circumference—WC, cm); (**b**)—percentage of participants with/without central obesity.

**Figure 4:**
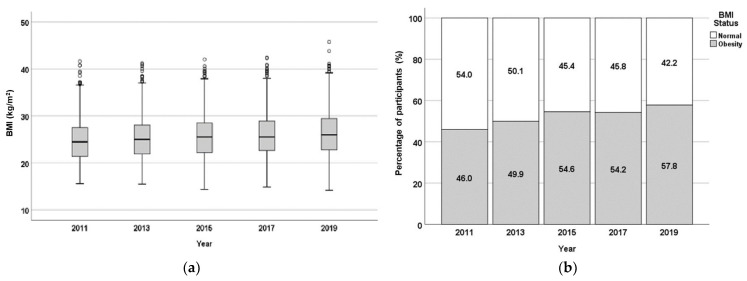
The BMI-based obesity status of the population of 867 respondents from FRTPM study in Indonesia during five years of observation (2011–2019), (**a**)—boxplot (BMI, kg/m^2^); (**b**)—percentage of participants with/without BMI-based obesity.

Central obesity is determined based on waist circumference (WC) (Figure 3). The average value of WC and the central obesity status (% of participants with identified central obesity) increased in the period under investigation, from 43.9% in 2011 to 69.9% in 2019. The obesity status based on BMI (Figure 4) also shows an increase in the percentage of obese subjects, from 43.9% in 2011 to 67.9% in 2019 (although there was a slight decrease between 2015 and 2017).

### 3.6. Observation of Biomedical Parameters of Non-Communicable Diseases

Biomedical parameters associated with non-communicable diseases, such as diabetes and cardiovascular disease, include glycemic control (fasting blood glucose/FBG and 2-h postprandial blood glucose), blood pressure (systolic and diastolic), and lipid profile (cholesterol, LDL-C, triglycerides, and HDL-C) [31]. The average values of biomedical parameters during the five years of observation in this study can be seen in Table 3 below.

The mean fasting blood glucose value ≥100 mg/dL was only recorded in 2017 (105.02 mg/dL) and 2019 (105.88 mg/dL), while blood glucose 2-h postprandial ≥140 mg/dL was only recorded in 2017 (144.39 mg/dL). The mean cholesterol levels exceeding the limit of 200 mg/mL were recorded in 2013 and 2019. The mean triglycerides (TG) values during the 5 years of observation have increased, but were still within normal limits (<150 mg/dL). The mean LDL cholesterol level during the 5 years of observation was nearly within the limits (100–128 mg/dL). The average HDL cholesterol concentrations during the 5 years of observation were all in the normal range (>40 mg/dL and <60 mg/dL). The LDL-C/HDL-C ratio during the 5 years of observation, except for 2017, exceeded 2.5, with the limit of 2.5 being recognized as an indicator of the risk of DM and cardiovascular disease [24]. In this study, both systolic and diastolic blood pressure were, on average, in the normal range during the 5 years of observation.

To summarize, the results obtained related to biomedical parameters in this study did not have the same pattern as changes in macronutrient intake, which tended to increase each year of observation.

## 4. Discussion

As previously mentioned, consumption patterns in the presented study showed that cereals and their processed products were the food group consumed in the greatest amount, by nearly 100% of respondents. A similar trend has previously been reported in other studies, focused on the European population [32]. Similarly, fats and oils belonged to the food groups consumed by almost all respondents in all study years, far exceeding the expected food pattern, which is also in agreement with other previous studies [33]. The observed vegetable and fruit consumption far below PPH also confirms global trends and proves the high importance of the intervention in this matter. The results on the energy and macronutrient intake were also mostly in agreement with other similar studies that reported protein and carbohydrate intake below the recommendations [34,35], the increased share of the population with fat intake exceeding nutritional adequacy [35], and the low fiber intakes, with almost 100% of the studied population not reaching adequate intake levels [36,37].

Similarly to the presented study, another study in Indonesia also reported that the main food groups that contribute to energy and protein intake in Indonesian households are cereals, especially rice, next to animal protein sources such as fish and chicken [38]. Another study in Indonesia stated that the main food groups that contribute to energy and protein intake in Indonesian households include carbohydrate staples and protein sources from food consumed outside homes, such as soups, satays, meatball noodles, cooked fish, and processed meat [39]. In another study, fish, poultry, red meat, eggs, milk, and plant sources such as cereals, nuts, and tubers were listed among the food groups that contributed the most to energy and protein intake in Indonesian households [40].

Research in Ireland has shown similar results to this study, reporting that cereal products make a significant contribution to the average daily intake of energy (26%), protein (21%), fat (13%), carbohydrates (41%), and fiber (45%) [41]. Food consumption survey data from five developed countries show that this food group contributes to the daily intake of energy, saturated fat, fiber, and certain nutrients with very similar percentages [42]. Research in the UK population also reported a large (47%) contribution of cereals and their processed foods to carbohydrate intake [43]. The results of this study and studies in several other countries show that the main food groups consumed in the country make the largest contribution to energy and nutrient intake. Other research in Indonesia reported that grain products provided the highest contribution to energy (67.2%) and protein (44.7%) consumption, while animal protein only contributed 38.7% [44].

Both the central obesity and the BMI-based obesity status (% of participants with identified obesity) increased in the period under investigation in the presented study, which was associated with excessive energy, carbohydrate, fat and protein intake and the fiber intake far below the RDA. A high-calorie and low-nutrient diet is known to contribute greatly to the incidence of obesity and NCDs [45], with the prevalence of these conditions significantly growing globally. Other studies also report that carbohydrate intake, especially from sweet foods, contributes significantly to obesity [7,46]. However, the increase in the obesity status in the present study is also suspected due to the increasing status of excess protein and fat intake each year of observation. The status of excessive protein intake in the subsequent years was as follows: 3.6%, 3.9%, 7.0%, 16.3%, 22.4%, while the status of excessive fat intake was 15.7%, 16.4%, 32.9%, 55.2%, 56.6%. Research in Australia has also shown an association between higher total protein intake and increased BMI and LP. Other studies have reported the associations between fiber intake and weight loss, improvement of lipid profiles, glucose metabolism, and blood pressure levels [47,48].

Even though excessive fat accumulation in the body is often attributed mainly to the energy intake exceeding energy expenditures, with excessive food consumption being identified as the primary cause of the imbalance, researchers also attribute the initial cause of obesity to an intrinsic metabolic defect that diverts fuel partitioning from pathways for mobilization and oxidation to pathways for synthesis and storage. The resulting reduction in fuel oxidation and energy capture in adipose tissue leads to a compensatory increase in energy intake [49]. It is generally believed that the primary cause of type 2 diabetes is obesity-induced insulin resistance in non-adipose tissues, combined with insufficient insulin secretion by pancreatic β-cells to overcome this resistance. High levels of circulating free fatty acids are deposited in insulin-sensitive non-adipose tissue cells, resulting in lip toxicity, which is an important cause of insulin resistance [50]. The increase in obesity status in this study can be an early indication of NCDs such as diabetes mellitus and CHD. This study found that excessive consumption patterns and nutrient intake, especially in fat, protein and carbohydrate intake, and lack of fiber intake, tended to increase obesity status, but did not show the same tendency in the diabetes mellitus and cardiovascular disease biomarkers such as blood glucose levels and lipid profile status (dyslipidemia). Even though not only consumption patterns and nutrient intake are risk factors for NCDs, obesity can trigger diabetes mellitus, which is associated with an increased risk of CHD [51].

Other studies also reported that obesity is not limited to fat accumulation, but there are other risk factors related to hormones as controllers of energy homeostasis. The concept of “disturbed energy balance” is believed to be the beginning of obesity, but various other factors, such as lack of sleep, lack of physical activity, and psychological factors, are also reported to have an influence on the development of obesity [50].

Macronutrient imbalance, particularly due to overnutrition, is a significant risk factor for the development of insulin resistance and type 2 diabetes mellitus. The intake of various nutrients, such as fructose, dietary fiber, fatty acids, and amino acids, clearly affects insulin sensitivity and glucose homeostasis. Adjusting the intake of these macronutrients is essential to prevent insulin resistance, highlighting the importance of understanding their role in energy balance and metabolic health in individuals with diabetes [52]. The relationship between macronutrient proportions and insulin resistance is complex and not fully understood. A high-fat, low-carbohydrate diet significantly reduces insulin resistance compared to a low-fat, high-carbohydrate diet [53]. Fat accumulation, particularly in the abdominal area, can increase insulinemia, which inhibits fat mobilization and oxidation. In individuals prone to obesity, reduced fat oxidation exacerbates this imbalance, contributing to insulin resistance. Thus, macronutrient imbalance, particularly regarding fat, plays a significant role in the development of insulin resistance [54].

Biomedical parameters associated with non-communicable diseases, such as diabetes and cardiovascular disease, reported in this study, included glycemic control, blood pressure, and lipid profile [31]. These biomedical markers did not show the same pattern as changes in macronutrient intake, which tended to increase each year of observation. This is in line with the results of other studies that did not show significant relationships between macronutrient intake and NCDs [55,56,57]. Interestingly, a study in China concluded that a decreasing trend in high carbohydrate intake, combined with an increasing trend in low fat intake, was significantly associated with an increased risk of diabetes among adults [58]. High daily calorie intake from carbohydrates, protein, and fat significantly contributes to the risk of complications in patients with type 2 diabetes mellitus. Carbohydrate intake exceeding 65% is associated with an increased risk of cardiovascular disease [59].

The cross-sectional epidemiological International Study of Macro/Micronutrients and Blood Pressure (INTERMAP), involving respondents from Japan, China, England and America, found that higher intake of vegetable protein and polyunsaturated fatty acids were inversely associated with blood pressure, while high intake of sugar and animal protein were directly associated with increased blood pressure levels [60]. A study in Korea reported that macronutrient composition significantly affects the risk of hypertension. An unbalanced diet that is high in carbohydrates and sodium is associated with an increased risk of hypertension, especially in women [61].

A Dietary Approaches to Stop Hypertension (DASH) study reported that a carbohydrate-rich diet, combined with an emphasis on fruits, vegetables, and low-fat dairy products and reduced saturated fat, total fat, and cholesterol, substantially lowered blood pressure and low-density lipoprotein cholesterol. The Optimal Macro-Nutrient Intake to Prevent Heart Disease (Omni Heart) study, showed that replacing some carbohydrates with protein (about half from plant sources) or with unsaturated fat (mostly monounsaturated fat) further reduced blood pressure, low-density lipoprotein cholesterol, and the risk of coronary heart disease. The results of these trials highlight the importance of macronutrients as determinants of cardiovascular disease risk [62].

A 6-year cohort study in Tehran showed an association between macronutrient quality, regardless of quantity, and the risk of chronic diseases, especially MetS [63]. Data from the Korean Genome and Epidemiology Study over 10 years found that a high glycemic load diet increased the risk of developing DM in middle-aged and older Korean men, but not in women [64]. Another 14-year cohort study concluded that higher energy, protein, and fat intake at dinner compared to breakfast increased the risk of DM [65].

The National Health and Nutrition Examination Survey (NHANES) in the United States, including a 15-year cohort study, reported that, in women, low fat (10%) and high carbohydrate (75%) consumption was associated with the least optimal TG and HDL-C. In men, HDL cholesterol was positively associated with fat and no association was detected with TG. The positive association of total cholesterol was especially in men in a diet consisting of 25% protein, 30% carbohydrate, and 45% fat. The highest positive association with systolic in both sexes was in a diet containing low fat (10%) combined with moderate protein (25%). The association with diastolic blood pressure was specific to women with higher values in those consuming fat in the upper range (55%). There was no association between macronutrient composition and glycemic control or adiposity. This study revealed a sex-specific association between macronutrient composition and cardiometabolic health. Further research is needed to explore this association across age groups [66].

A 17-year longitudinal study found that higher diet quality scores were associated with lower risk of MetS or its components among Tehran adults. Higher intake of healthy food group components and lower consumption of unhealthy food group components of the diet quality score predicted lower incident MetS and its risk factors [67]. In a prospective study over 18 years, a higher intake of plant protein was associated with lower total mortality and cardiovascular mortality. Although animal protein intake was not associated with mortality outcomes, replacing red meat or processed meat protein with plant protein was associated with lower total mortality, cancer-related mortality, and cardiovascular mortality [68]. However, another study that also took 18 years, concluded that there was no association between low carbohydrate and high protein consumption with cardiovascular disease [69]. Results of an 18-year study in China showed that both high and low carbohydrate percentages were associated with an increased risk of new-onset hypertension, with minimal risk at 50% to 55% carbohydrate intake. The increased risk was mainly found in those with a low intake of high-quality carbohydrates or a high intake of low-quality carbohydrates. These findings support high-quality carbohydrate intake, and replacement of low-quality carbohydrates with plant products, for hypertension prevention [70]. The 32-year Nurses’ Health Study I, and 26-year Nurses’ Health Study II, reported that carbohydrate quality plays an important role in the risk of type 2 diabetes. High-quality carbohydrates, especially those from whole grains, were associated with a lower risk of type 2 diabetes. Conversely, low-quality carbohydrates, such as those from refined grains and added sugars, were associated with a higher risk of type 2 diabetes [71].

The results of several studies described above suggest that explanation of the relationships between consumption patterns and NCDs and/or their respective biomarkers requires a long observation period, and taking into consideration the complexity of lifestyle and sociodemographic characteristics, as well as the complexity of dietary factors including, among others, the balance of energy and individual macronutrients but also their quality and dietary sources.

The presented study provides important insights into diet-related risks for obesity and non-communicable diseases in Indonesia, but it has its limitations. The self-reported 24-h dietary recall applied in the study, while valuable for assessing dietary intake, can be affected by recall bias and misreporting, and does not fully allow for capturing all habitual intake characteristics and for the identification of irregularly consumed foods in a typical diet. The accuracy of the undertaken estimations could therefore be enhanced by multiple 24-h recalls or validated food frequency questionnaires. Moreover, the adjustment of the outcomes for a number of potential lifestyle and sociodemographic factors, unexecuted in the study, could allow for further exploration and explanation of the observed trends.

Further research should consider the larger sample size, consumption data in the form of multiple 24-h recall or/and validated food frequency questionnaire, monitoring of a complexity of lifestyle and sociodemographic factors, the addition of other NCD risk factor variables, and, last but not least, a longer observation period.

## 5. Conclusions

To summarize, the study identified cereals as the food group consumed in the largest amount and the largest contributor to energy, protein, carbohydrates and fiber intake. The fats and oils group exceeded the recommended intake, while vegetable and fruit consumption, and consequently the fiber intake, were far below the recommendations, with almost 100% of the studied population not reaching adequate fiber intake levels.

The energy and macronutrient intake, and the percentage of respondents with excessive intake levels, were consequently increasing during the studied years. The consumption pattern of food groups, both in terms of type and quantity, and consequently specific macronutrients, was associated with the incidence of obesity, with the main indications being identified as high consumption of food groups that strongly contributed to overall energy, fat and carbohydrate intake, and fiber consumption below the RDA. At the same time, the biomedical parameters (NCDs biomarkers) measured remained relatively stable despite increasing obesity and macronutrient intake over the study period.

The presented study provides important insights into diet-related risks for obesity in Indonesia. Its results could inform public health policies and help design intervention strategies in Indonesia, such as developing targeted dietary education campaigns, to address excessive fat and insufficient fiber intake.

## Figures and Tables

**Table 1:** Food consumption patterns of the population of 867 respondents from FRTPM study in Indonesia during five years of observation (2011–2019).

Food Groups	2011		2013		2015		2017		2019	
	n	Mean ± SD (g)	n	Mean ± SD (g)	n	Mean ± SD (g)	n	Mean ± SD (g)	n	Mean ± SD (g)
Cereals and cereal products	867	259.61 a ± 111.36	865	241.47 b ± 91.97	866	259.11 c ± 104.41	867	255.41 c ± 113.86	866	234.84 d ± 132.06
Starchy roots and tubers and products	389	16.33 a ± 31.95	298	16.51 b ± 37.33	526	24.66 a ± 45.26	543	34.03 c ± 52.13	598	43.37 c ± 65.82
Legumes, nuts and seeds, and products	690	76.02 a ± 76.60	743	67.39 b ± 58.18	756	83.79 c ± 78.71	751	94.14 d ± 84.36	737	83.29 e ± 78.10
Vegetables and products	742	48.13 a ± 42.46	726	48.72 b ± 47.29	747	49.71 c ± 46.19	773	68.08 d ± 58.81	782	61.15 e ± 52.77
Fruits and products	420	73.19 a ± 107.21	436	50.56 b ± 77.21	370	55.29 c ± 96.13	482	85.88 a ± 121.56	477	82.39 b ± 122.16
Meat and products	505	42.56 a ± 51.89	541	36.44 b ± 43.76	525	50.36 c ± 74.85	579	63.75 d ± 69.21	644	71.68 e ± 76.41
Finfish, shellfish, other aquatic animals and products	464	25.34 a ± 36.44	465	27.30 b ± 37.33	491	32.15 c ± 55.48	430	26.23 d ± 42.85	388	22.43 e ± 41.31
Eggs and products	470	29.93 a ± 40.70	594	36.10 b ± 40.18	464	30.82 c ± 41.65	516	35.73 d ± 44.30	577	37.22 e ± 45.99
Milk and products	145	6.27 a ± 26.69	74	3.57 b ± 20.08	101	6.78 c ± 37.11	325	10.83 d ± 46.95	384	13.13 e ± 43.59
Fats and oils	862	41.71 a ± 38.74	858	37.05 b ± 40.28	862	55.04 c ± 48.67	861	71.05 d ± 55.84	862	78.62 e ± 71.68
Sugars, syrup and confectionery	641	19.55 a ± 18.10	506	17.19 b ± 33.06	658	21.95 b ± 22.95	691	25.61 bc ± 27.09	722	34.19 c ± 26.48
Spices and condiments	859	11.43 a ± 10.33	850	12.58 b ± 13.81	864	15.12 c ± 17.78	866	19.66 a ± 21.50	860	13.47 d ± 15.05
Alcoholic beverages	-	-	-	-	1	0.21 a ± 6.28	2	0.27 b ± 7.65	4	1.51 c ± 43.47
Nonalcoholic beverages	735	205.70 a ± 231.26	788	421.15 b ± 357.41	791	426.35 c ± 369.04	802	442.09 d ± 347.95	794	471.06 e ± 411.44
Fast foods	1	0.12 a ± 3.40	2	0.10 b ± 2.17	2	0.23 c ± 4.80	3	0.33 d ± 7.79	5	0.76 e ± 10.46
Mixed food dishes	301	17.01 a ± 33.70	477	31.47 b ± 45.63	274	12.21 c ± 28.57	358	17.94 d ± 31.81	372	19.72 e ± 43.89
Miscellaneous	-	-	-	-	-	-	-	-	3	0.00 a ± 0.08
Traditional herbs and supplements	6	0.01 a ± 0.11	14	0.30 b ± 4.05	10	0.04 c ± 0.70	25	0.33 d ± 3.52	27	0.27 e ± 4.87
Total		872.92		1047.90		1123.82		1251.34		1 69.11 e
Drinking water	853	984.97 a ± 537.14	831	1005.10 a ± 598.61	853	1229.13 c ± 630.26	855	1297.88 d ± 657.42	855	1413.01 e ± 749.08
Total with drinking water		1857.89		2052.99		2352.94		2549.22		2682.12

Different letters within the same row (a-e) show statistically significant differences between years (Wilcoxon Paired Test, *p* < 0.05).

**Table 2:** Food group contributions to energy, macronutrients and fiber intake per capita/day of the population of 867 respondents from FRTPM study in Indonesia during five years of observation (2011–2019) (mean ± SD).

Food Groups	Energy Contribution		Protein Contribution		Fat Contribution		Carbohydrate Contribution		Fiber Contribution	
	kcal/Day	%	g/Day	%	g/Day	%	g/Day	%	g/Day	%
Cereals and products	868.43 ± 389.20	47.71 ± 13.75	20.96 ± 9.50	37.20 ± 13.98	5.04 ± 3.40	9.21 ± 8.11	176.63 ± 79.96	71.57 ± 14.42	1.70 ± 2.08	28.03 ± 22.83
Starchy roots, tubers and products	33.75 ± 81.15	1.78 ± 3.97	0.34 ± 0.69	0.63 ± 1.79	0.40 ± 2.24	0.57 ± 2.62	7.24 ± 16.57	2.92 ± 6.16	0.23 ± 0.71	3.82 ± 9.50
Legumes, nuts and seeds, and products	125.60 ± 127.12	6.81 ± 6.11	10.75 ± 10.14	18.02 ± 14.86	7.06 ± 8.20	10.56 ± 10.23	7.22 ± 8.50	3.06 ± 3.49	1.04 ± 1.23	17.82 ± 17.70
Vegetables and products	16.28 ± 16.35	0.92 ± 0.94	0.99 ± 1.11	1.76 ± 2.10	0.26 ± 0.37	0.44 ± 0.65	3.05 ± 3.12	1.31 ± 1.37	1.17 ± 1.59	18.54 ± 17.17
Fruits and products	48.84 ± 74.86	2.66 ± 3.99	0.58 ± 0.91	1.05 ± 1.75	1.08 ± 4.27	1.53 ± 5.47	11.86 ± 18.06	4.79 ± 7.02	1.43 ± 2.64	17.67 ± 22.97
Meat and products	150.13 ± 191.12	7.79 ± 9.04	11.51 ± 15.12	16.94 ± 18.14	11.03 ± 14.73	14.88 ± 16.75	0.92 ± 3.14	0.38 ± 1.33	0.01 ± 0.14	0.21 ± 2.27
Finfish, shellfish, other aquatic animals and products	34.66 ± 58.00	1.97 ± 3.09	5.89 ± 10.23	9.56 ± 13.07	0.81 ± 2.03	1.43 ± 3.26	0.78 ± 2.06	0.33 ± 0.88	0.00 ± 0.00	0.00 ± 0.07
Eggs and products	56.38 ± 71.16	3.12 ± 4.04	4.06 ± 5.24	7.11 ± 9.33	3.63 ± 4.69	5.75 ± 7.83	1.47 ± 5.86	0.63 ± 2.25	0.01 ± 0.06	0.26 ± 1.40
Milk and products	17.49 ± 51.44	0.93 ± 2.70	1.11 ± 3.06	1.8 ± 4.70	0.69 ± 2.64	1.01 ± 3.89	1.71 ± 4.83	0.73 ± 2.12	0.00 ± 0.00	0.00 ± 0.00
Fats and oils	321.26 ± 237.12	16.78 ± 8.16	0.50 ± 0.96	0.82 ± 1.59	35.43 ± 26.32	50.69 ± 17.46	1.82 ± 3.57	0.73 ± 1.44	0.34 ± 0.67	5.29 ± 10.01
Sugars, syrup and confectionery	85.28 ± 97.58	4.76 ± 5.15	0.29 ± 1.30	0.49 ± 2.10	0.40 ± 1.68	0.65 ± 2.76	19.43 ± 21.11	8.04 ± 8.15	0.07 ± 0.55	0.78 ± 5.05
Spices and condiments	8.05 ± 13.29	0.44 ± 0.66	0.60 ± 2.01	0.96 ± 1.82	0.10 ± 0.25	0.16 ± 0.41	1.18 ± 1.72	0.52 ± 0.83	0.11 ± 0.34	2.06 ± 5.14
Alcoholic beverages	0.80 ± 34.03	0.01 ± 0.52	0.00 ± 0.13	0.00 ± 0.09	0.00 ± 0.00	0.00 ± 0.00	0.03 ± 1.28	0.01 ± 0.23	0.00 ± 0.00	0.00 ± 0.00
Nonalcoholic beverages	27.55 ± 68.20	1.52 ± 3.63	0.73 ± 3.42	1.22 ± 3.46	0.12 ± 0.56	0.24 ± 1.31	4.62 ± 12.88	1.92 ± 5.12	0.19 ± 1.25	2.42 ± 8.56
Fast foods	0.87 ± 18.17	0.05 ± 1.09	0.07 ± 1.58	0.09 ± 1.89	0.05 ± 1.21	0.08 ± 1.71	0.02 ± 0.57	0.01 ± 0.34	0.00 ± 0.04	0.02 ± 0.77
Mixed food dishes	48.98 ± 88.27	2.78 ± 5.03	1.30 ± 2.48	2.33 ± 4.51	1.67 ± 3.34	2.78 ± 5.68	7.23 ± 13.51	3.10 ± 5.77	0.17 ± 0.48	3.07 ± 8.09
Miscellaneous	-	-	-	-	-	-	-	-	-	-
Traditional herbs and supplements	0.01 ± 0.87	0.001 ± 0.09	0.001 ± 0.06	0.002 ± 0.14	-	-	0.003 ± 0.18	0.01 ± 0.36	-	-
Drinking water	-	-	-	-	-	-	-	-	-	-
Total	1844.36	100.30	59.68	99.99	67.77	99.98	245.20	100.05	6.49	100.00

**Table 3:** Biomedical parameter (selected NCDs biomarker) values of the population of 867 respondents from FRTPM study in Indonesia during five years of observation (2011–2019) (mean ± SD).

Parameters	Year				
	2011	2013	2015	2017	2019
Fasting blood glucose (mg/dL)	92.04 ± 25.91	85.80 ± 24.25	95.73 ± 30.29	105.02 ± 44.80	105.88 ± 38.90
Blood glucose 2-h postprandial (mg/dL)	126.05 ± 53.66	123.23 ± 45.87	136.34 ± 55.04	144.39 ± 68.04	138.48 ± 63.25
Total cholesterol (mg/dL)	195.70 ± 34.81	200.23 ± 35.15	196.47 ± 35.78	199.13 ± 36.73	204.82 ± 38.54
Triglycerides (mg/dL)	106.96 ± 62.79	117.25 ± 71.05	116.73 ± 68.08	122.59 ± 78.52	128.60 ± 87.40
LDL cholesterol (mg/dL)	126.49 ± 30.49	128.09 ± 31.75	128.03 ± 32.65	123.64 ± 31.59	128.27 ± 33.54
HDL cholesterol (mg/dL)	51.37 ± 11.46	52.14 ± 12.35	52.89 ± 11.25	51.80 ± 12.35	48.86 ± 11.89
LDL-C/HDL-C ratio	2.58 ± 0.82	2.56 ± 0.82	2.52 ± 0.81	2.50 ± 0.82	2.75 ± 0.93
Blood pressure, systolic (mmHg)	129.79 ± 23.78	127.29 ± 22.29	127.80 ± 21.42	104.83 ± 29.54	128.01 ± 20.09
Blood pressure, diastolic (mmHg)	81.34 ± 13.24	80.76 ± 12.72	80.34 ± 12.05	83.97 ± 12.15	81.00 ± 11.08

## Data Availability

Data will be made available upon request by the author Nuri Andarwulan.
